# Papillary thyroid cancer in prepubertal patients: A report of two cases and a brief review of the literature 

**DOI:** 10.3892/mi.2025.273

**Published:** 2025-10-01

**Authors:** Abdulwahid M. Salih, Aras J. Qaradakhy, Ari M. Abdullah, Karzan M. Salih, Shaho F. Ahmed, Zana B. Najmadden, Meer M. Abdulkarim, Harun Amanj Ahmed, Shko H. Hassan, Abdullah A. Qadir, Aso N. Qadir, Fahmi H. Kakamad

**Affiliations:** 1 Department Scientific of Affairs, Smart Health Tower, Sulaymaniyah 46001, Iraq; 2 College of Medicine, University of Sulaimani, Sulaymaniyah 46001, Iraq; 3 Department of Radiology, Shorsh Teaching Hospital, Sulaymaniyah 46001, Iraq; 4 Department of Pathology, Sulaymaniyah Teaching Hospital, Sulaymaniyah 46001, Iraq; 5 Research Center, University of Halabja, Halabja 46018, Iraq; 6 Zad Organization, Sulaymaniyah 46001, Iraq; 7 Kscien Organization for Scientific Research (Middle East Office), Sulaymaniyah 46001, Iraq

**Keywords:** papillary thyroid carcinoma, thyroidectomy, radioiodine therapy, cancer

## Abstract

Although papillary thyroid carcinoma (PTC) is relatively rare in children, particularly in the prepubertal age group, its biological behavior resembles that of other pediatric solid tumors, where delayed diagnosis can significantly affect outcomes. In the broader oncology context, rare pediatric malignancies, such as prepubertal PTC, highlight the importance of tailoring diagnostic and therapeutic strategies to those applied in adults. PTC in prepubertal children is a rare occurrence that presents with a high likelihood of distant metastasis. The present study describes 2 cases of PTC in 9-year-old girls with irrelevant medical, surgical, or family history. The first case was that of a 9-year-old girl with a painless neck swelling for 4 weeks. An ultrasonography revealed diffuse thyroid lesions and cervical lymphadenopathy. She underwent a total thyroidectomy with bilateral neck dissection. Pathological analysis confirmed diffuse sclerosing PTC with numerous positive lymph nodes. She recovered well and received radioactive iodine therapy. The second case was that of a 9-year-old girl with right-sided neck swelling for 1 year. Imaging revealed a Thyroid Imaging Reporting and Data System 5 nodule and suspicious lymph nodes. She underwent a total thyroidectomy with bilateral central and right lateral neck dissection. Pathological analysis confirmed unifocal conventional PTC with 17 positive lymph nodes. She recovered well, received radioactive iodine therapy, and remained recurrence-free for 3 years. In addition, in the present study, a literature search was performed on the PubMed and Google Scholar database, covering the period from January, 2017 to February, 2025. The search used combinations of the following key words: ‘papillary thyroid carcinoma’, ‘papillary thyroid cancer’, ‘thyroid neoplasm’, ‘pediatric’ and ‘prepubertal’. Only articles published in the English language and reporting individual prepubertal PTC cases were considered. From this search, 9 relevant cases were identified and were included in a brief review of the literature, of which 5 cases were females. The ages of the patients ranged from 5 to 17 years. The right thyroid lobe was involved in 4 patients, and bilateral involvement was reported in 1 case. Tumors were multifocal in 7 patients, and lymph nodes were involved in 8 patients. A total of 7 cases were of conventional variation, and distant metastases were reported in 4 cases, all of which were in the lungs. Total thyroidectomy was performed for all the cases. As demonstrated herein, papillary thyroid carcinoma in prepubertal children is uncommon. Total thyroidectomy may provide good long-term outcomes.

## Introduction

Thyroid tumors are uncommon among the pediatric population, representing ~0.7% of all childhood cancers (1). Despite this rarity, thyroid cancer is the most prevalent type of endocrine malignancy among children, and its incidence increases with age, reaching its peak between 15 and 19 years of age (2). Among the different types of thyroid cancer, papillary thyroid carcinoma (PTC) is the most frequently diagnosed, accounting for ~80-90% of all pediatric thyroid cancer cases (3). Although PTC is rare in children, it often presents with more extensive lymph node involvement and a higher likelihood of distant metastases than in adults, making early detection and appropriate treatment crucial.

Several risk factors have been found to be associated with the development of thyroid cancer, including Hashimoto's thyroiditis, genetic disorders such as multiple endocrine neoplasia type 2, Carney's syndrome, Werner's syndrome and DICER1 syndrome, iodine deficiency, as well as exposure to ionizing radiation, particularly during childhood (1). These risk factors highlight the importance of the careful monitoring of individuals with relevant medical histories. Pediatric thyroid cancer is often initially discovered as a neck mass, typically without accompanying symptoms, which can result in a range of progression stages at the time of diagnosis. Although rare, thyroid cancer in children can be easily mistaken for other non-thyroid conditions, such as abscesses, malformations, ectopic thymus, thyroglossal duct cysts and various tumors. This misdiagnosis can lead to delays in appropriate treatment, highlighting the importance of considering thyroid cancer in the differential diagnosis of pediatric neck masses (2).

PTC subtypes include classic, solid, follicular and diffuse sclerosing variants. In children, particularly those aged <10 years, the classic papillary morphology often observed in adults may be absent. These tumors can be unencapsulated and widely invasive throughout the thyroid, displaying a follicular and solid architecture with unique nuclear features and abundant psammoma bodies (4).

The present study describes two rare cases of PTC in pediatric patients with no notable medical or family history of thyroid cancer, both of whom underwent total thyroidectomy without complications. Furthermore, the present study aimed to contribute valuable information to the current body of literature through a detailed review of the existing information. The present case report was written in accordance with the CaReL guidelines. Only reliable, peer-reviewed sources were used while excluding any untrustworthy references or data (5,6).

## Case report

### Case 1. Patient information

A 9-year-old girl presented to the Head and Neck Clinic at Smart Health Tower (Sulaymaniyah, Iraq) with a painless anterior neck swelling that had been present for four weeks. She had no significant family, medical, or surgical history.

*Clinical findings*. The patient was vitally stable. Upon examination, the thyroid gland was firm and enlarged, with palpable cervical lymph nodes.

**Figure 1:**
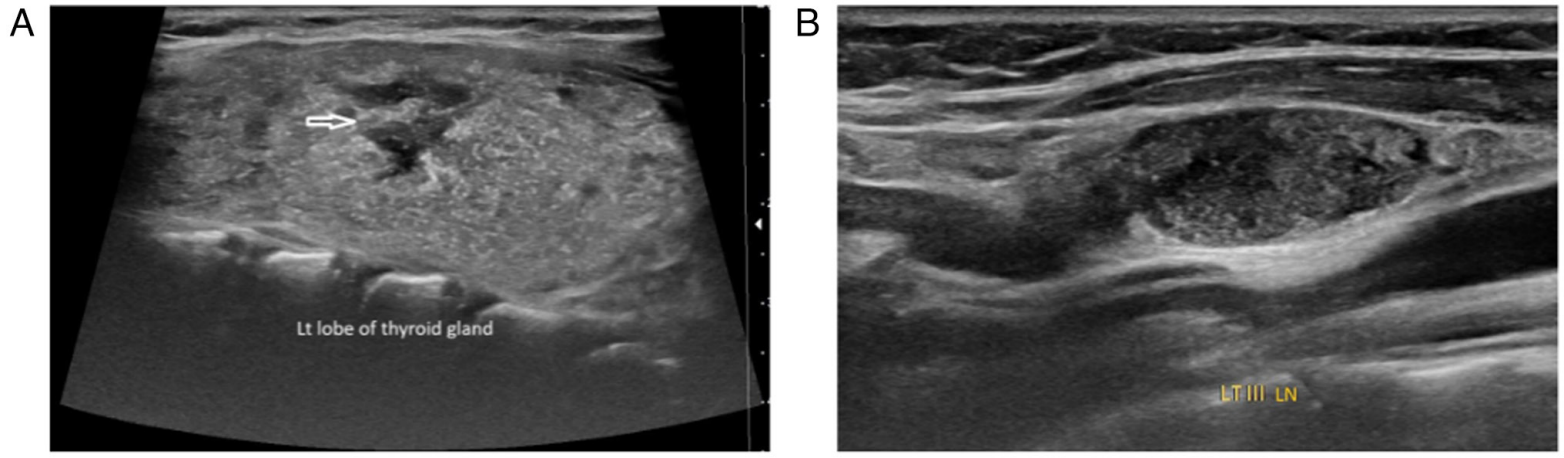
(A) Ultrasound image of case 1 illustrating an enlarged left lobe of the thyroid gland with heterogeneous parenchymal echotexture, irregular margins, hypoechoic lesions (arrow), and diffuse scattered microcalcifications in both lobes. (B) Ultrasound image of case 1 demonstrating multiple bilateral lateral cervical lymph nodes with features highly suspicious for malignancy.

*Diagnostic approach*. Laboratory investigations revealed normal thyroid function. The thyroid-stimulating hormone (TSH) level was 3.3 uIU/ml (normal range, 0.8-6.0 uIU/ml), the calcitonin was 0.724 pg/ml (normal range, up to 9.82 pg/ml), the anti-thyroglobulin level was elevated at 373 IU/ml (normal range, <115 IU/ml) and the serum calcium level was normal at 9.62 mg/dl (normal range, 8.8-10.8 mg/dl). A neck ultrasound (US) revealed a mildly enlarged thyroid gland with heterogeneous parenchymal texture, and multiple irregular hypoechoic lesions were noted in both lobes, primarily on the left and diffuse microcalcifications in both lobes. Suspicious lymph nodes were identified around the gland, the largest measuring 17x8 mm. They were also identified in the right cervical groups I, II, III and IV, the largest measuring 22x6 mm, and the left groups II, III, IV, and V, the largest measuring 20x9 mm (Fig. 1). Fine-needle aspiration cytology (FNAC) under ultrasound guidance was suggestive of PTC.

**Figure 2:**
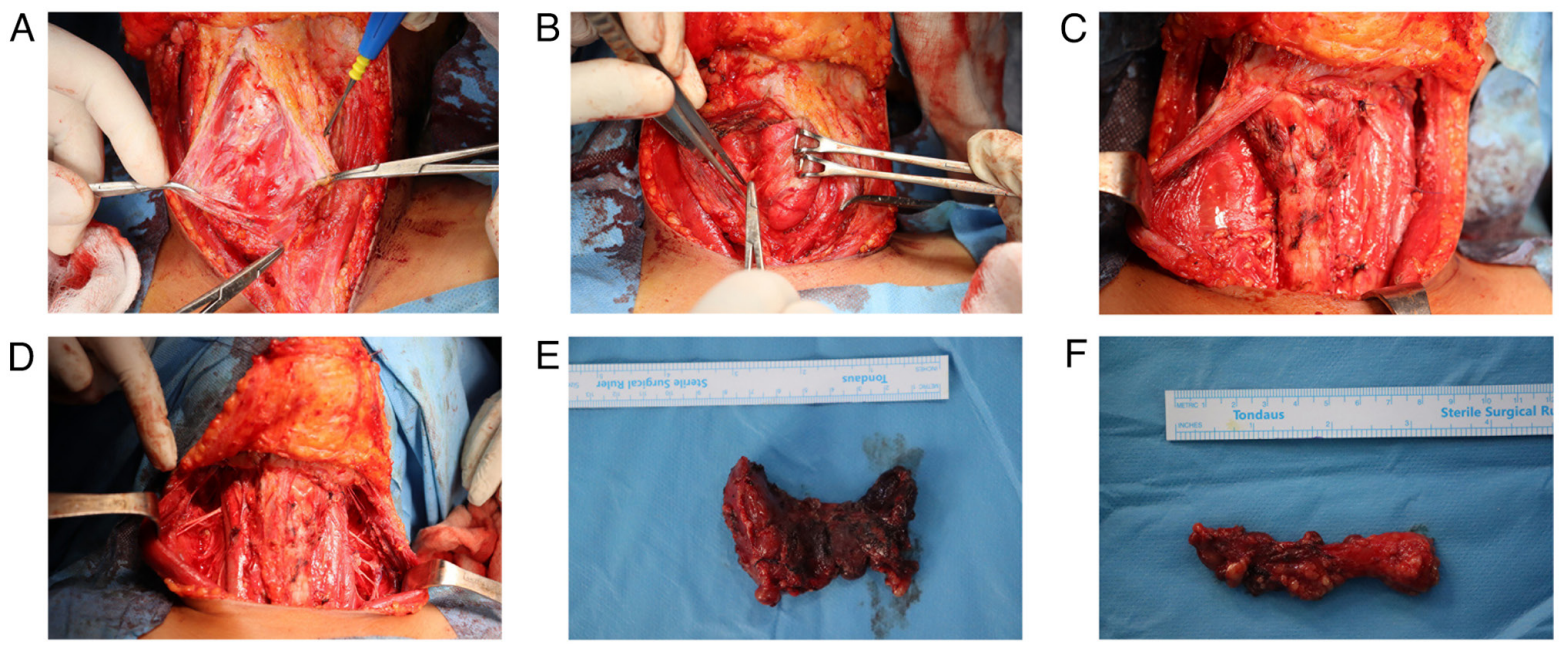
(A) Initial exposure of the anterior neck of case 1 following the elevation of subplatysmal flaps and midline separation of strap muscles. (B) Careful dissection and identification of the recurrent laryngeal nerve in the tracheoesophageal groove. (C) Mobilization of the thyroid gland with exposure of central neck structures and surrounding compartments. (D) Completion of total thyroidectomy and bilateral central and lateral (levels I-V) neck dissection. Both recurrent laryngeal nerves were preserved. (E) Gross image of the resected thyroid specimen showing an irregular, nodular surface consistent with multifocal papillary thyroid carcinoma. (F) Excised lymph node specimen from the lateral neck compartment prepared for histopathological assessment.

**Figure 3:**
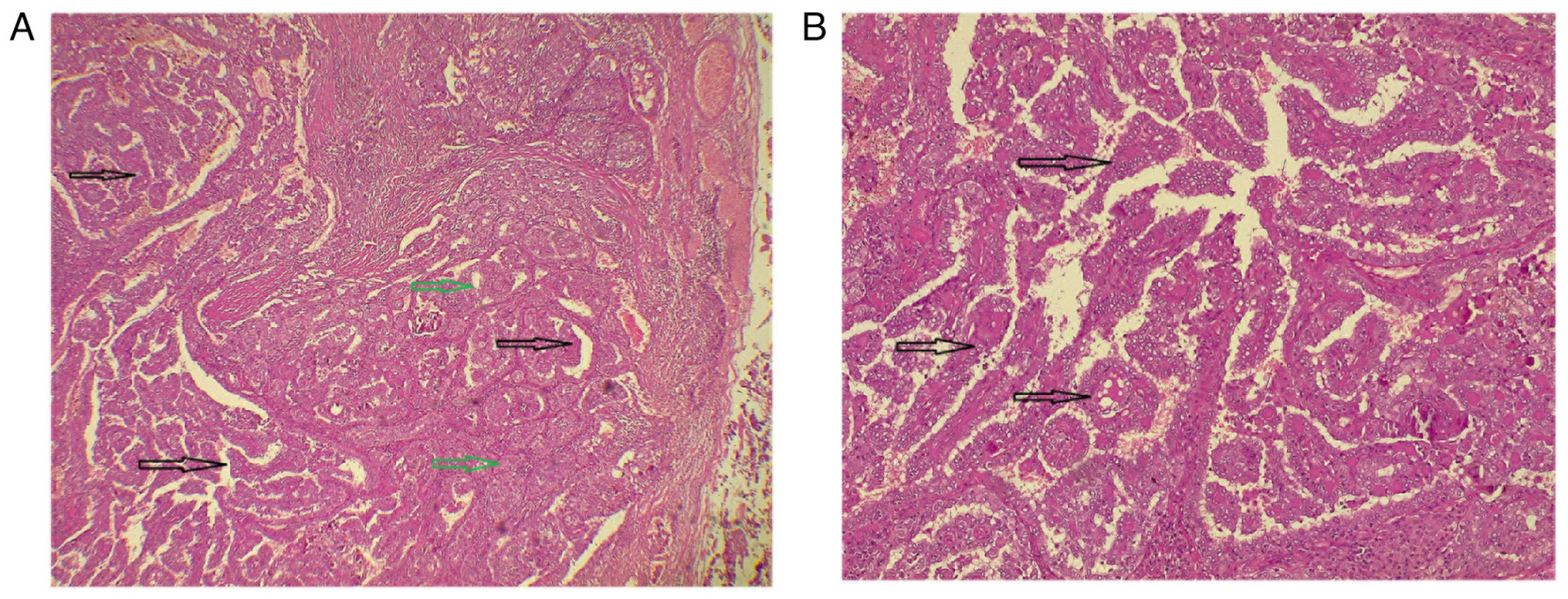
(A) Lower-power view of a section from the first case, demonstrating complex papillary structures (black arrows) with limited follicle formation (green arrows) infiltrating the stroma (hematoxylin and eosin staining; magnification, x4). (B) Higher-power view showing papillary structures with fibrovascular cores (black arrows), lined by follicular epithelial cells exhibiting optically clear nuclei and irregular nuclear membranes (hematoxylin and eosin staining; magnification, x10).

*Therapeutic intervention*. Under general anesthesia, the patient underwent a total hyroidectomy with bilateral central and lateral neck dissection (levels I to V). Both recurrent laryngeal nerves were preserved, hemostasis was achieved, and the wound was closed in layers (Fig. 2). Post-operatively, the patient remained stable. A histopathological examination (HPE) was performed. Tissue samples were fixed in 10% neutral-buffered formalin at room temperature for 24 h, processed and embedded in paraffin. Sections of 5 µm thickness were prepared, stained with hematoxylin and eosin (Bio Optica Co.) for 1-2 min at room temperature, and subsequently examined using a light microscope (Leica Microsystems GmbH). The HPE revealed a multifocal, well-differentiated diffuse sclerosing type PTC involving the right lobe, isthmus and left lobe, with the largest lesion measuring 55 mm in the left lobe (Fig. 3). Of the 181 lymph nodes examined, 70 were positive for metastasis, including the Delphian lymph nodes (9/9), right central group (8/9), left central group (5/14), right lateral group (6/45) and left lateral group (8/104).

*Follow-up*. The patient recovered without complications and was discharged (at almost 1 week following her presentation to the hospital). with prescribed thyroid medication and a scheduled follow-up appointment. She was referred for radioactive iodine treatment and will have regular follow-ups to monitor for any signs of recurrence.

### Case 2. Patient information

A 9-year-old girl presented to the Head and Neck Clinic at Smart Health Tower (Sulaymaniyah, Iraq) with right-sided neck swelling that had been gradually enlarging over the past year. She had no accompanying symptoms or notable medical, surgical, or family history. There was also no history of irradiation or consanguinity.

*Clinical findings*. Upon examination, the thyroid gland was firm and enlarged at the right lobe, with palpable cervical lymph nodes.

**Figure 4:**
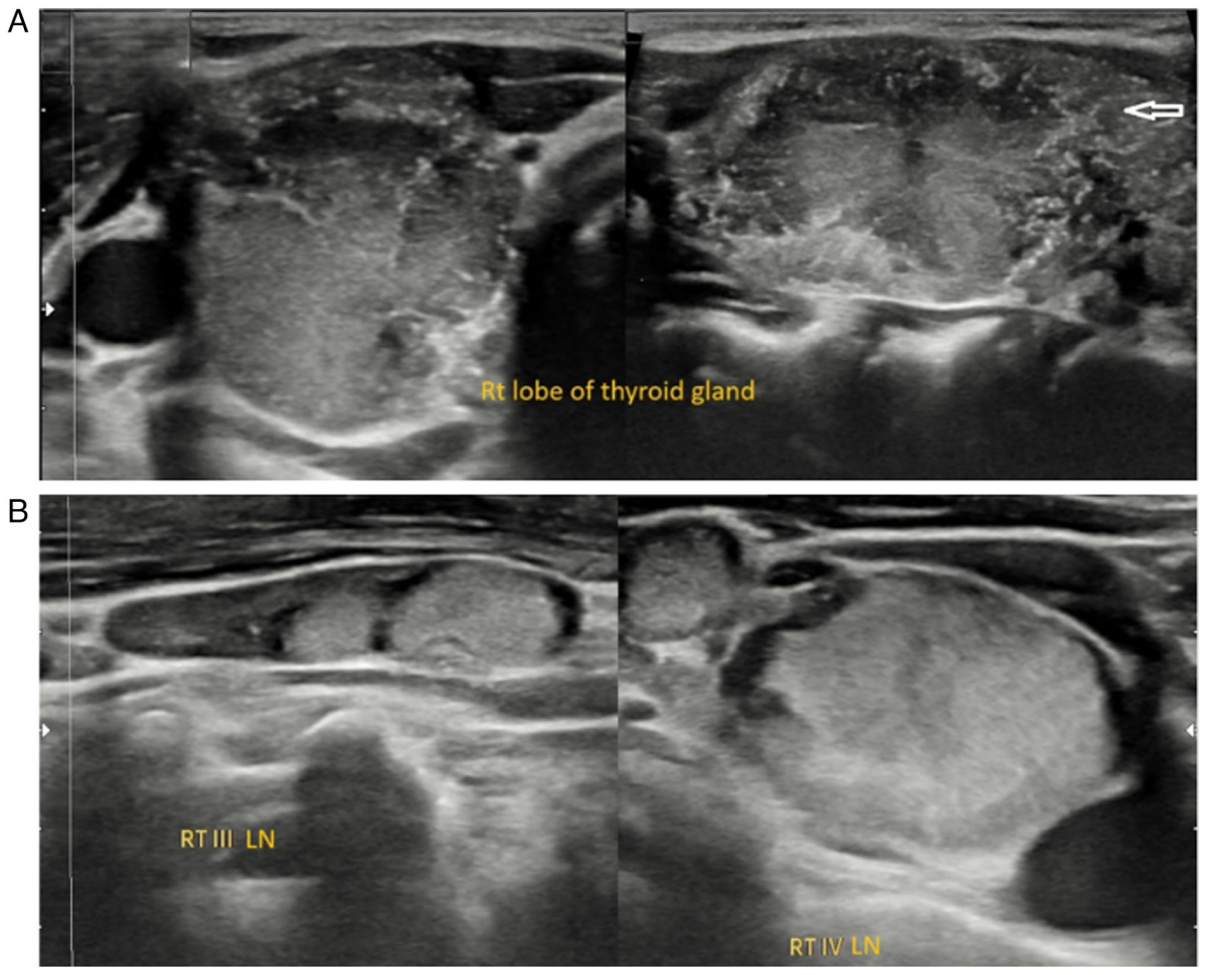
(A) Ultrasound image of case 2 of the right thyroid lobe demonstrating a large solid hypoechoic nodule measuring 34x19x19 mm with irregular margins and internal microcalcifications, some of which extend beyond the nodule margins (arrow). (B) Ultrasound image of case 2 illustrating bilateral lateral cervical lymph nodes with features highly suspicious for malignancy.

*Diagnostic approach*. Upon a laboratory investigation, thyroid function tests were normal; the TSH level was 2.77 uIU/ml (normal range, 0.8-6.0 uIU/ml), the free T4 level was 16.62 Pmol/l (normal range, 12.8-27 Pmol/l), the calcitonin level was 3.56 pg/ml (normal range, up to 9.82 pg/ml), the thyroglobulin level was 2.53 ng/ml (normal range, 3.5-77 ng/ml) and the normal serum calcium level was 9.35 mg/dl (normal range, 8.8-10.8 mg/dl). The neck US revealed a large, irregular outline, hypervascular, heterogenously hypoechoic nodule measuring 37x19x19 mm, occupying the majority of the right lobe, mainly mid-upper third with microcalcification, classified as a highly suspicious nodule Thyroid Imaging, Reporting And Data System (TI-RADS) 5(7). This was associated with suspicious pathological cervical lymph nodes in the right group II, III and IV, the largest measuring 24x5 mm. In the left thyroid lobe, there were a few small micronodules (Fig. 4). The patient underwent FNAC under US guidance for the right TR5 nodule, and the cellular findings confirmed PTC.

**Figure 5:**
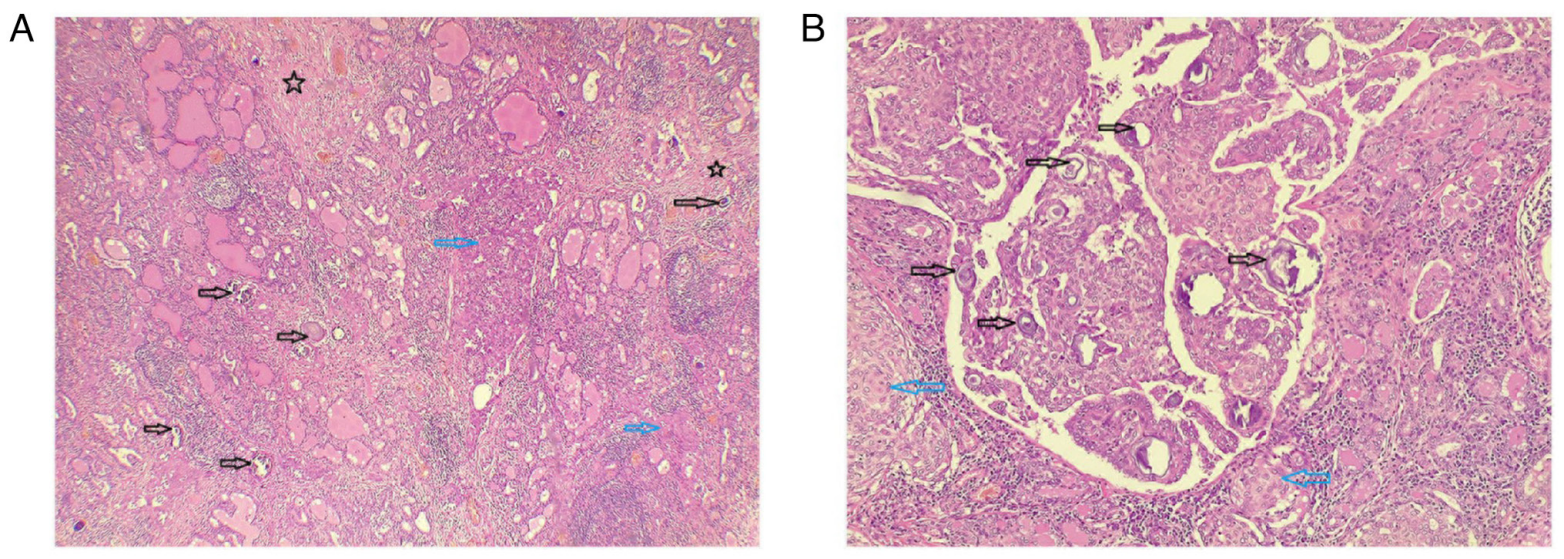
(A) Low-power view of thyroid tissue (case 2) demonstrating multiple foci of stromal fibrosis (black stars), solid and loosely cohesive epithelial cell sheets (blue arrows) and numerous psammoma bodies (black arrows); hematoxylin and eosin staining; magnification, x4. (B) High-power view (case 2) illustrating nests and sheets of malignant epithelial cells with nuclear features of papillary thyroid carcinoma, including solid areas (blue arrows), along with numerous psammoma bodies (black arrows); hematoxylin and eosin staining; magnification, x40.

**Figure 6:**
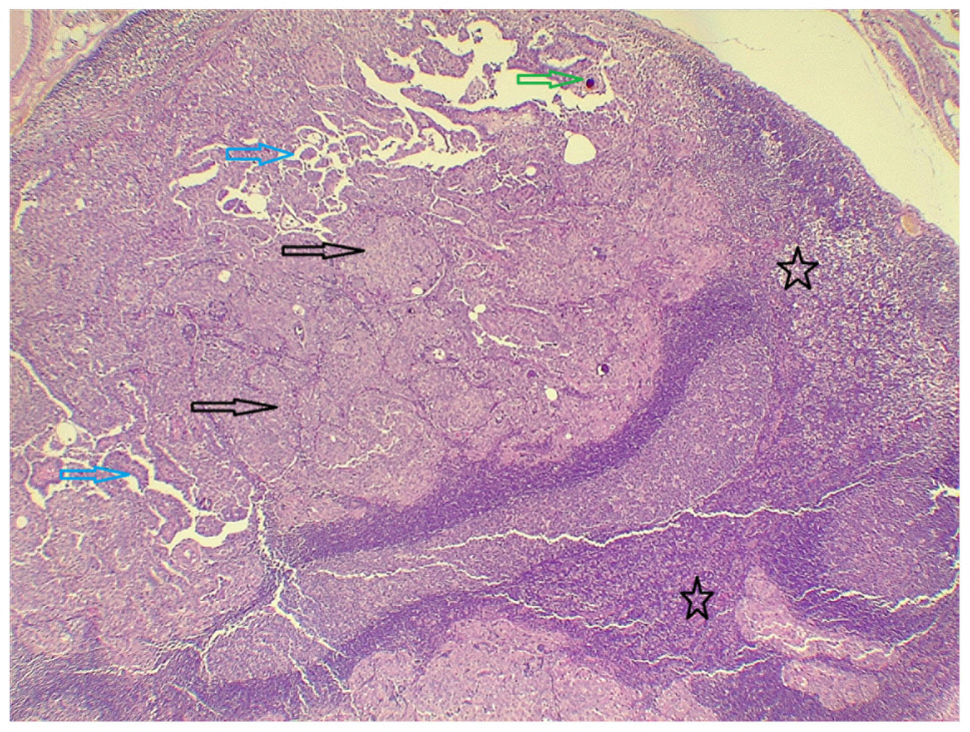
Low-power view of a cervical lymph node composed of benign lymphoid tissue with reactive follicles (case 2; black stars), infiltrated by malignant epithelial cells forming papillary structures (blue arrows) and solid areas (black arrows), with psammoma bodies also present (green arrow); hematoxylin and eosin staining; magnification, x4.

*Therapeutic intervention*. Following a thorough discussion within a multidisciplinary team, the patient underwent a total thyroidectomy with bilateral central and right lateral neck dissection under general anesthesia. Both recurrent laryngeal nerves were preserved. Hemostasis was achieved and the wound was closed in layers. The post-operative course was uneventful, with stable vital signs. A HPE (performed as described above for Case 1) confirmed a unifocal, well-differentiated conventional PTC in the right lobe (Fig. 5). Lymph node involvement was noted in 17 out of 72 nodes, which exhibited infiltration by malignant epithelial cells forming papillary structures: Delphian (0/2), right central (8/15), left central (1/17) and right lateral (8/38) (Fig. 6).

*Follow-up*. The patient exhibited an uneventful postoperative recovery and was discharged (at almost 1 week following her presentation to the hospital) on thyroid hormone replacement therapy with a scheduled follow-up. She was referred for adjuvant radioactive iodine therapy and enrolled in a structured surveillance program to monitor for disease recurrence. There was no clinical or radiological evidence of recurrence at the three-year follow-up.

## Discussion

PTC, particularly among prepubertal patients, is considered rare. When compared with adult PTC, pediatric patients with PTC often present with relatively more advanced-stage tumors with a notable female predominance. However, pediatric patients with PTC, if properly treated, have an improved prognosis compared with older patients with PTC (2,8). This was evident in the patients described herein, as the first case exhibited multifocality, with the largest lesion measuring 55 mm, accompanied by metastasis in 70 lymph nodes. The second case also exhibited signs of local advancement as the tumor was 37 mm in size, and 17 lymph nodes had been invaded. A literature search was performed on the PubMed and Google Scholar database, covering the period from January, 2017 to February, 2025. The search used combinations of the following key words: ‘papillary thyroid carcinoma’, ‘papillary thyroid cancer’, ‘thyroid neoplasm’, ‘pediatric’ and ‘prepubertal’. Only articles published in the English language and reporting individual prepubertal PTC cases were considered. From this search, 9 relevant cases were identified and were included in a mini-review of the literature (2,3,8-14). In line with the current body of literature and indicative of advancement, 7 out of 9 (77.77%) cases exhibited multifocality, and 8 (88.88%) cases had lymph node involvement (Table I).

The most common sites of metastasis are the lungs, bones and brain. Long-distance metastasis was present in 4 (44.44%) of the reviewed cases, all in the lungs. The primary treatment for lung metastases in thyroid cancer is radioactive iodine therapy, which can achieve complete radiographic resolution and provide long-term survival benefits for patients (9). The most significant predictors of recurrence are lymph node involvement, multiple thyroid nodules at presentation, and papillary or papillary-follicular histology. Recurrence rates are higher in children (35-45%) compared to adults (5-20%) (2). In the present study, among the reviewed cases, 4 patients (44.44%) experienced recurrence, all with lymph node involvement. Thyroid cancer is generally more common among females than males, with an overall ratio of ~1 male to every 3.6 females. However, in children aged <10 years, this difference becomes less pronounced, with a male-to-female ratio of ~1.25:1. The incidence of thyroid cancer peaks between the ages of 15 and 19 years, with the average age at diagnosis being 16 years (2). The patients in the present case report were both 9 years of age, and the mean age of diagnosis was 11±3.84 for the reviewed cases.

The majority of children with PTC are typically diagnosed after noticing symptoms such as an enlarging thyroid nodule or a persistent neck lymph node. The diagnostic process often involves a physical exam, thyroid US, fine-needle biopsy, and, if deemed necessary, a diagnostic hemithyroidectomy (10). A distinct subtype, pediatric diffuse sclerosing PTC, such as that observed in case 1 in the present study, is characterized by extensive infiltration, resulting in enlargement of the affected thyroid lobe or the entire gland, often with palpable cervical lymphadenopathy. This variant is frequently associated with microcalcifications, making FNAC essential for definitive diagnosis (3). The primary imaging modality for evaluating neck swelling in children is ultrasonography. Features suggestive of thyroid malignancy include hypoechogenicity, an irregular outline, a subcapsular location and type III nodular vascularization (both peri-nodular and intra-nodular), which are strongly associated with an increased likelihood of malignancy in pediatric patients. FNAC remains the cornerstone of the diagnostic workup for thyroid nodules in children, providing a minimally invasive and reliable method for evaluating malignancy risk (15). The 2009 ATA guidelines for adult thyroid cancer recommend staging all patients with differentiated thyroid carcinoma (DTC) according to the AJCC TNM classification. Within this framework, children are categorized as stage I if no distant metastases are present and stage II if distant metastases exist (4). Notably, the stage I group is highly heterogeneous, encompassing children with a solitary intrathyroidal lesion, those with extensive locoregional disease and cervical lymph node involvement, as well as those with only microscopic PTC (4).

In recent years, it has become widely accepted that a total thyroidectomy is necessary for all pediatric PTC cases, along with the surgical removal of any affected lymph nodes when feasible, as it has been found that the absence of total thyroidectomy is one of the most significant risk factors for recurrence (10). A point of surgical debate is whether to perform a central compartment lymph node dissection, as it is associated with a higher risk of hypoparathyroidism. This risk needs to be carefully balanced against the potential for disease progression. Preserving the recurrent laryngeal nerves, as accomplished in both cases, is crucial, as damage can lead to vocal cord paralysis. Therefore, the surgery needs to be performed by experienced professionals (10). Children generally have a longer life expectancy than adults; thus, it is crucial to carefully evaluate the long-term impacts of thyroid cancer treatment in pediatric patients. Radioiodine therapy, while effective, is associated with risks of complications, such as dysfunction of the salivary and lacrimal glands (the most common side effects), reductions in fertility for both males and females, bone marrow suppression and an increased risk of developing secondary cancers (3). A lower socioeconomic status and diagnostic delays can limit access to timely care and specialized diagnostics, leading to more advanced disease at presentation with greater nodal involvement and the need for aggressive treatment. In pediatric PTC, where multifocality and nodal spread are common, such factors further increase recurrence risk and complicate management.

Future advances in PTC should rely on precision medicine with molecular profiling for targeted therapy, AI-assisted diagnostics to improve early detection, and liquid biopsy for non-invasive monitoring. Studying heterogeneous nuclear ribonucleoprotein C is also critical, as its expression levels have been linked to tumor mutational burden (16). Selective radioactive iodine and minimally invasive surgery support treatment de-escalation in patients who are considered low-risk, while global collaborations are vital to reduce disparities and refine pediatric-specific guidelines that balance survival with long-term quality of life.

A key limitation of the present study was the absence of comprehensive genetic or molecular profiling, such as assessment of BRAF, RAS, RET/PTC, or other kinase fusions, which are increasingly recognized as critical for risk stratification, prognosis and targeted therapy selection in pediatric PTC. The lack of this information limits the ability to associate molecular alterations with clinical outcomes and hinders personalized treatment planning.

In conclusion, PTC in prepubertal children is uncommon. Total thyroidectomy with adjuvant radioiodine therapy may provide favorable outcomes; however, management needs to be carefully individualized, and current evidence is limited to small case series, underscoring the need for larger studies.

## Figures and Tables

**Table I:** Fundamental characteristics of the reviewed cases.

Age, years	Sex	Med. history	Family history	Location	Size in rad. (mm)^a^	Multifocality	Lymph node involvement	Hist. variant	Mutations	Dist. Met.	Surgical approach	Radioactive iodine therapy	Other adjuvant therapy	Recurrence	Outcome	Follow-up, years	(Refs.)
11	F	Autoimmune thyroiditis	Hypothyroidism and vitiligo	Right thyroid lobe	45	Yes	Yes	Diffuse sclerosing	N/A	None	Total thyroidectomy and central compartment lymphadenectomy	Yes	Suppressive levothyroxine therapy	None	Alive	0.3	(3)
8	M	Unremarkable	Unremarkable	Right thyroid lobe	100	Yes	Yes	Conventional	N/A	None	Total thyroidectomy	No	Thyroxine	None	Alive	4	(2)
15	F	β-Thalassemia, acute pancreatitis, transaminitis, Fanconi-like syndrome & secondary amenorrhea	Unremarkable	Right thyroid lobe	59	Yes	Yes	Conventional	N/A	None	Total thyroidectomy and central compartment neck dissection	Yes	Levothyroxine	Yes	N/A	2	(11)
17	M	Unremarkable	N/A	Thyroglossal duct	65	No	No	Conventional	N/A	None	Extended Sistrunk procedure and total thyroidectomy	No	Thyroid Hormone Suppression Therapy	No	Alive	0.5	(12)
5	M	Peripheral cyanosis	N/A	N/A	20	No	Yes	Conventional	N/A	Lungs	Total thyroidectomy & cervical lymph node excision	Yes	None	Yes	Died	5.5	(13)
11	F	Unremarkable	N/A	N/A	N/A	Yes	Yes	Diffuse sclerosing	N/A	Lungs	Total thyroidectomy & lymph node dissection	N/A	N/A	N/A	Alive	N/A	(10)
12	M	Hyperthyroidism	N/A	N/A	N/A	Yes	Yes	Conventional	N/A	Lungs	Total thyroidectomy	Yes	None	Yes		0.5	(9)
13	F	Unremarkable	Thyroid carcinoma	Right thyroid lobe	40	Yes	Yes	Conventional	N/A	Lungs	Total thyroidectomy and bilateral central compartment lymph node excision	Yes	None	Yes	N/A	N/A	(14)
7	F	Unremarkable	N/A	Bilateral lobes of the thyroid	R=35 L=18	Yes	Yes	Conventional	ETV6/NTRK3 fusion	None	Total thyroidectomy and bilateral cervical lymph node dissections	Yes	None	No	Alive	N/A	(8)
Mean: 11±3.84	F: 5/9 M:4/9	5/9	2/9			7/9	8/9	C: 8/9 D: 1/9	1/9	4/9	TT: 9/9	6/9	4/9	4/9	A: 5/9 D: 1/9		

^a^Only the largest dimensions are presented to keep the heterogeneity of data presentation. Med. history, medical history; Size in rad., size in radiology; Hist. variant, histological variant; Dist. met, distant metastasis; F, female; M, male; N/A, not applicable; R, right; L, left; C, conventional; D, diffuse sclerosing; TT, total thyroidectomy; A, alive; D, deceased.

## Data Availability

The data generated in the present study may be requested from the corresponding author.
